# Radioresistance of Human Cancers: Clinical Implications of Genetic Expression Signatures

**DOI:** 10.3389/fonc.2021.761901

**Published:** 2021-10-27

**Authors:** Sven de Mey, Inès Dufait, Mark De Ridder

**Affiliations:** Department of Radiotherapy, Universitair Ziekenhuis Brussel, Vrije Universiteit Brussel, Brussels, Belgium

**Keywords:** radiotherapy, TCGA (The Cancer Genome Atlas Program), gene set analysis, prognostic, head and neck (H&N) cancer, cervical cancer, breast cancer

## Abstract

Although radiotherapy is given to more than 50% of cancer patients, little progress has been made in identifying optimal radiotherapy - drug combinations to improve treatment efficacy. Using molecular data from The Cancer Genome Atlas (TCGA), we extracted a total of 1016 cancer patients that received radiotherapy. The patients were diagnosed with head-and-neck (HNSC - 294 patients), cervical (CESC - 166 patients) and breast (BRCA - 549 patients) cancer. We analyzed mRNA expression patterns of 50 hallmark gene sets of the MSigDB collection, which we divided in eight categories based on a shared biological or functional process. Tumor samples were split into upregulated, neutral or downregulated mRNA expression for all gene sets using a gene set analysis (GSEA) pre-ranked analysis and assessed for their clinical relevance. We found a prognostic association between three of the eight gene set categories (Radiobiological, Metabolism and Proliferation) and overall survival in all three cancer types. Furthermore, multiple single associations were revealed in the other categories considered. To the best of our knowledge, our study is the first report suggesting clinical relevance of molecular characterization based on hallmark gene sets to refine radiation strategies.

## Introduction

Radiotherapy (RT) represents an essential treatment modality in cancer management, either alone or combined with other therapies. Approximately 50% of all cancer patients will receive radiotherapy at some time in their illness, resulting in a cure rate of about 40% (1). Over the past few decades, technological advances and clinical research have given radiation oncologists the capability to personalize treatments for accurate delivery of radiation dose based on clinical parameters and anatomical information (2). However, individual responses to RT vary widely among disease types and patient populations (3). The resistance to RT is associated with several biological alterations of the tumor cells and the tumor microenvironment (4). Unravelling the processes and hallmarks of cancer cells that lead to radioresistance will provide critical insights for future research into combination therapies with radiotherapy.

A detailed understanding of the cellular pathways involved in the response to irradiation is imperative to pave the way to more individualized RT. The last 20 years, precision medicine has harnessed genetic profiling’s power to personalize cancer treatments, nonetheless similar predictions regarding tumor benefit following RT are lagging (5). Prior studies have examined associations between genotype and clinical radiosensitivity; DNA repair pathway alterations such as deleterious germline and somatic mutations in genes such as *BRCA1, BRCA2, PALPB2,* and *ATM* have been studied extensively as potential biomarkers of radiation sensitivity (6). Next to this, several clinically relevant associations have been described between epigenetics, hormone axis, receptor tyrosine kinase (RTK) signaling, intracellular signaling, the tumor microenvironment and radioresistance (7). Although these studies often identify genomic features that are known to be prognostic, translating these findings into actionable treatment decisions remains a significant challenge (7, 8).

To comprehensively evaluate clinically relevant genetic signatures, which may function as therapeutic targets of different cancer types, we integrated and analyzed clinical information and mRNA-sequencing data from head-and-neck (HNSC), cervical (CESC) and breast cancer (BRCA) patients that underwent radiotherapy. First, we selected the 50 hallmark gene expression signatures from the molecular signature database collection (MSigDB) (9). Each hallmark in this collection consists of a “refined” gene set, derived from multiple “founder” sets, that conveys a specific biological state or process and displays coherent expression. Using the comprehensive molecular and clinical data compiled in The Cancer Genome Atlas (TCGA) (10), we associated the upregulation or downregulation of these 50 hallmark gene expression signatures with the patients’ clinical outcome. This approach allowed to divide the patients in the three different groups based on their respective gene expression levels (upregulated, neutral or downregulated) and correlated these groups with the patients’ clinical outcome. To the best of our knowledge, our study is the first report suggesting clinical relevance of molecular characterization based on hallmark gene sets to refine radiation strategies.

## Results

### Patient Characteristics

To investigate the clinically relevant molecular features of cancers with regard to survival following radiotherapy, we selected three relevant cancers: head-and-neck squamous cancer (HNSC), cervical squamous cancer (CESC), and breast (BRCA) cancer. HNSC comprises the sixth leading cancer diagnosis and includes a heterogeneous group of malignant tumors arising in all structures of the head-and-neck region, except for the brain, spinal cord, skull base, and skin. Patients with limited disease (T1-2N0) are treated by surgery or radiotherapy, according their general condition and functional implication of the treatment. Patients with locally advanced HNSC undergo surgery, followed by adjuvant chemoradiotherapy or radiotherapy alone. If these patients are not amenable to surgery they are treated by primary (chemo)radiation (11). CESC is the fourth most common female malignancy worldwide (12). Low stage CESC patients are treated with either surgery or RT. Concurrent chemotherapy with RT is given as adjuvant therapy for high-risk stages I to IIA. In stages IB, IIA-B, III, and IVA, concurrent chemotherapy with RT is given as primary therapy. For both HNSC and CESC the prognostic impact of tumor oxygenation during radiotherapy has been described in several retrospective studies. BRCA is a highly heterogeneous disease and the most common neoplasm in women. Although classic histopathologic classification of breast cancer remains important, molecular characterization of the disease is rapidly emerging as a vital tool for understanding clinical prognosis. Noninvasive and early invasive BRCA are usually treated with breast conserving surgery following adjuvant RT. Depending on TNM-stage, hormone receptor markers, HER2-status, differentiation grade, proliferation markers, … are early invasive BRCA patients also treated with (neo)adjuvant radio(chemo)therapy (11). We chose these cancer types to offer a general overview of the 50 hallmark gene sets’ influence on survival of patients who underwent RT.

We extracted data from all the 299 HNSC, 168 CESC and 549 BRCA patients that underwent radiotherapy in The Cancer Genome Atlas (TCGA) Pan-Cancer cohort. Their characteristics are shown in **Table 1**. Human Papillomavirus (HPV) negative (-) was the most common subtype in HNSC patients. At the same time, most of the CESC were squamous carcinomas and the majority of BRCA patients had a Luminal A subtype. The patient’s age ranged from 19 to 90, with the HNSC patients being the oldest (median 59.34), followed by the BRCA patients (median 56.71) and the CESC patients (median 48.89). The majority of patients were female and of the white race. All genetic data was taken from patients after they underwent surgery, but before the patients underwent adjuvant (chemo)radiotherapy. RT treatment schedules were as follows; HNSC patients received a median of 62Gy with a treatment duration of 45 days. CESC patients received a median of 40Gy with 31.5 days of treatment and BRCA patients received 60Gy with a treatment time of 43 days. BRCA patients displayed the most extended overall survival (OS) and disease-free survival (DFS) (median OS: 32.48, DFS: 31.3), followed by CESC patients in OS (26.01) and DFS (26.79) time and HNSC patients presented with the shortest survival time (OS: 22.59, DFS: 22.42).

**Table 1:** Patient characteristics.

	HHSC	CESC	BRCA
**No. of cases**	**299**	**168**	**549**
**Subtype**			
**HPV-**	**228 (76.8)**		
**HPV+**	**50 (16.7)**		
**Adeno Carcinoma**		**23 (13.7)**	
**Squamous carcinoma**		**131 (78)**	
**Basal**			**89 (10.7)**
**HER2**			**33 (6)**
**LumA**			**253 (46.1)**
**LumB**			**98 (17.9)**
**Normal**			**17(3.1)**
**HA**	**21 (7)**	**14 (8.3)**	**59 (10.7)**
**Age (mean +-SOf**	59.34 +- 10.64	**48.89+-14.28**	**56.71 +- 12.13**
**Sex M/F**	**231/68**	**0/168**	**4/545**
**Stage**			
**I**	**8(2.7)**		**92 (16.8)**
**II**	**10 (6)**		**280 (51)**
**III**	**36 (12.7)**		**160 (29.1)**
**IV**	**184(61.5)**		**8 (1.5)**
**X**			**6 (1.1)**
**HA**	**51 (17.1)**	**168 (100)**	**3 (0.5)**
**TNM**			
**T1/T2/T3/T4/TX/TIS/NA**	**22/60/57/113/34/0/13**	**56/48/16/5/11/1/31**	**149/301/84/14/1/0/0**
**N0/N1/N2/H3/NX/NA**	**76/42/122/2/43/14**	**54/36/0/0/47/31**	**220/192/78/52/7/0**
**M0/M1/MX/NA**	**129/0/45/125**	**53/8/75/32**	**453/9/87/0**
**Ethnicity**			
**Hispanic or latino**	**13 (43)**	**10 (6)**	**25 (4.6)**
**Hot hispanic or latino**	**265 (88.6)**	**78 (46.4)**	**427 (77.8)**
**NA**	**21 (7)**	**80 (47.6)**	**97 (17.7)**
**Race**			
**American Indian or Alaska native**	**2 (0.7)**	**1 (0.6)**	**1 (0.2)**
**Asian**	**6 (2)**	**5 (3)**	**10 (1.8)**
**Black, or African American**	**28 (9.4)**	**17 (10.1)**	**98 (17.9)**
**White**	**256 (85.6)**	**123 (732)**	**398 (72.5)**
**HA**	**7 (2.3)**	**22 (13 1)**	**42 (7.7)**
**Dose (Gy) (median –IQR)**	**62 (60 - 66)**	**40 (27 - 45)**	**60 (50 – 60.4)**
**Treatment days (median-IQR)**	**45 (41 – 52)**	**31.5 (18.75 - 42.25)**	**43 ( 32 - 47)**
**Overall survival** **(Months) (median-IQR)**	**22.59 (13.69 - 41.72)**	**26.01 (16.17 - 48.67)**	**32.48 (18.6 – 63.3)**
**Disease free survival Months (median -IQR)**	**22.42 (12.92 - 41.85)**	**26.79 (16.83 - 53.59)**	**31.3 (18.21 - 59.67)**

### Genetic Expression Subtypes Show Clinically Relevant Patterns

**Figure 1:**
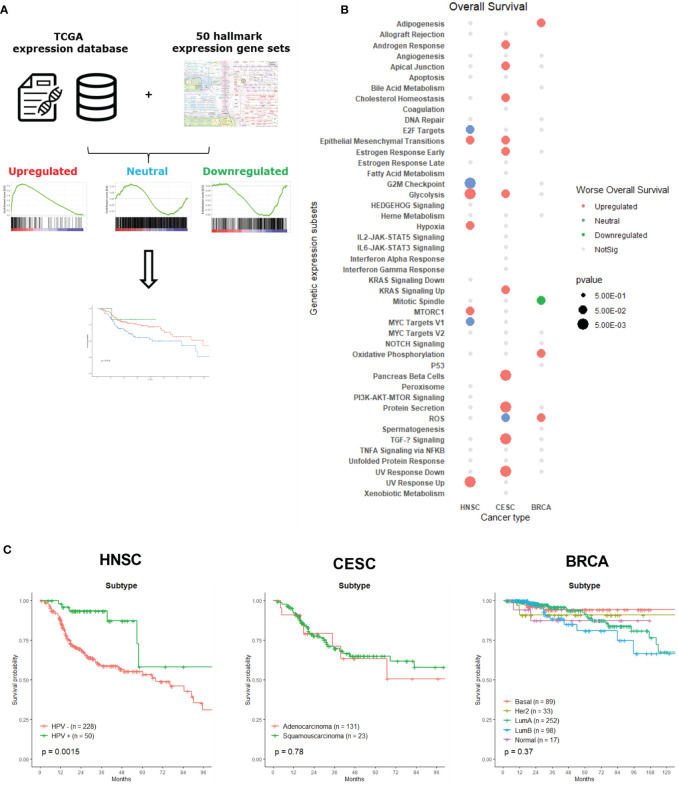
Classification and association of gene set expression signatures with patient survival times and tumor subtypes. **(A)** The computational method to classify tumor samples into three expression subtypes: upregulated, neutral and downregulated. These subtypes were than associated with patient OS. **(B)** Clinical associations of gene set expression signatures with patient OS. Color indicates correlation direction and size the level of p-value (log-rank test). Grey dots were not significant and had p-value lower than 0.5. Blanc spots were not significant for association with OS and had a p-value higher than 0.5. **(C)** Kaplan-Meier plots for molecular subtypes of HNSC, CESC and BRCA associated with patient overall survival times.

To assess the heterogeneity of the different hallmark expression gene sets across the three cancer types, we used the computational method developed by Peng et al. (13). First, we extracted the z-score ranked RNAseq data of each tumor sample for the above-described cancer patients. Z-scored data is RNAseq data normalized across samples by Z score to obtain a rank value for each gene. Next, we conducted a GSEA analysis, using the hallmark expression gene sets, on the resulting rank values to classify tumors into three subtypes: upregulated, neutral or downregulated (14, 15). The method derives its power by focusing on gene sets, that is, groups of genes that share a common biological function, chromosomal location, or regulation. Tumor samples for which the hallmark expression genes showed enrichment with high Z scores were defined as an upregulated subtype. In contrast, samples showing the opposite pattern were defined as downregulated subtype. Samples belonged to the neutral subtype when they did not show a significant enrichment pattern. Note that the concept of “upregulated” or “downregulated” here is relative to other tumor samples within the same cancer type, rather than relative to normal tissues (**Figure 1A**). We divided the 50 hallmark expression gene sets into eight categories based on their shared overlapping biological or functional process, namely Radiobiological, Metabolic, Proliferation, Development, Signaling, Cellular component, Pathway and Immune (**Supplementary Table 1**). We are aware that certain gene sets can be allocated into different categories, for example the G2M Checkpoint gene set into the proliferation category instead of the Radiobiological category, as genes often exert their functions on multiple levels. The eight described categories were based upon the proposed division of MSigDB and subsequently adapted to our own notion (9).

To assess the clinical relevance of the expression subtypes identified above, we determined associations with patient’s OS using a Kaplan – Meier analysis, since survival represents a critical clinical index of tumor aggressiveness. **Figure 1B** shows the summary of 24 significant survival associations for the 50 hallmark expression gene sets associated with one of the three cancer types. Only 45 of the 50 hallmark gene sets are depicted, since there were no survival associations for the Apical Surface, Complement, Inflammatory response, Myogenesis and WNT Beta Catenin Signaling gene sets. At first glance, CESC patient’s survival was associated with more hallmark gene sets than the other two cancer types. Most of the significant associations across cancer types are related to upregulated gene sets. Since we look for a link between these different gene signatures and survival, we cannot exclude that we are measuring intrinsic aggressiveness of the tumors, sensitivity to chemotherapy or other features that can influence the prognosis of the patients, next to radiosensitivity. However, the hallmark gene sets represent specific well-defined biological states or processes and display coherent expression. Also, this gene set collection has been thoroughly validated (9).

Specific tumor subtypes are identified in a clinical setting, as those tumor subtypes that are informative about cancer pathophysiology and are, in some cases, used for clinical decision making. For example, targeted therapies based on molecular targets such as BCR-ABL inhibitors in leukemia (16), BRAF and MEK inhibitors in melanoma (17, 18) and therapies targeting epidermal growth factor receptor (EGFR) in lung cancer and head-and-neck cancer patients (19) have significantly impacted the cancer treatment landscape. Therefore, we first examined whether these known clinical tumor subtypes influenced the survival of patients who underwent radiotherapy. **Figure 1C** shows the survival curves of each tumor type per cancer. In our analysis, only a statistically significant difference between HPV- and HPV+ HNSC patients could be observed. HPV+ cancer patients who underwent radiotherapy displayed a better survival than HPV- negative cancer patients, which was already demonstrated in recent research (20, 21). There was no significant survival difference between adeno- or squamous-carcinoma CESC patients, which is not surprising considering the small number of patients with squamous cell carcinoma. The absence of a difference between the molecular BRCA cancer subtypes may be explained by the variety of specific adjuvant treatment strategies that are delivered ranging from anti-hormonal therapy and anti-HER2-antibodies to (dose-dense) chemotherapy.

### Radiobiological Category

**Figure 2:**
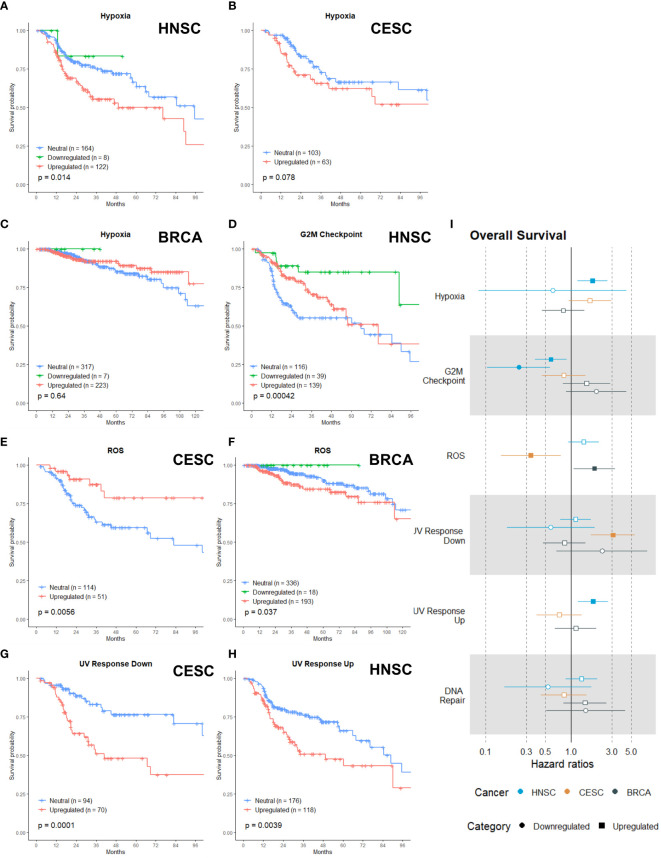
Associations of gene sets of the Radiobiological category with patient overall survival times. Kaplan-Meier plots for the Hypoxia gene set of **(A)** HNSC, **(B)** CESC and **(C)** BRCA patients associated with OS. **(D)** Kaplan-Meier plot for the G2M Checkpoint gene set of HNSC patients associated with OS. Kaplan-Meier plots for the ROS gene set for **(E)** CESC and **(F)** BRCA patients associated with OS. **(G)** Kaplan-Meier plot for the UV Response Down gene set for CESC patients associated with OS. **(H)** Kaplan-Meier plot for the UV Response Up gene set for HNSC patients associated with OS. **(I)** Forest plot showing the results from CoxPH model fits for OS of all the Radiobiological category gene sets. Results for the HNSC patients are in blue, brown for the CSC patients and black for the BRCA patients. Upregulated gene sets are depicted with a □ symbol [closed ■ means these results are significant (p>0.05)]. Downregulated gene sets are depicted with a ○ symbol [closed ● means these results are significant (p>0.05)].

The radiobiological category incorporated six gene sets, namely: Hypoxia, G2M Checkpoint, Reactive Oxygen Species (ROS), UV Response Down, UV Response Up and DNA Repair. We clustered these gene sets because each of them has a direct link with radioresistance. Already since the 1950s, it was known that hypoxic cancer cells were more resistant to ionizing radiation. Measured by polarographic oxygen electrode, the oxygenation status in CESC, HNSC and soft tissue sarcomas has been proven to be an adverse prognostic factor for radiotherapy (22, 23). These measurements were performed in tumors that were treated with primary radiotherapy. According to our analysis, in a post-operative setting, upregulation of the hypoxia gene set had a negative association with OS for HNSC (**Figure 2A**, p = 0.014) and a negative (non-significant) trend for CESC (**Figure 2B**, p = 0.078) patients, but not for BRCA patients (**Figure 2C**, p = 0.64). Surprisingly, was there an association for the HNSC and CESC patients and not for the BRCA patients, since all three patients groups were irradiated postoperatively to eliminate lingering cancer cells, which evidently are not subjected to hypoxic conditions.

In general, cells are the most sensitive to radiotherapy in the G2 or M phase of the cell cycle (24–27). Successful research has been performed by combining inhibitors of the G2M checkpoint arrest with radiotherapy (28–32). Only for HNSC patients, the upregulation and downregulation of the G2M gene set was associated with better survival (**Figure 2D**, p=0.00042). HNSC patients with an upregulated G2M gene had a survival benefit for the first two years; afterwards the survival curve converged with the curve of the reference (neutral) patient group (**Figure 2D**).

ROS causes approximately two-thirds of radiation-induced DNA damage, so cancer cells’ capability to detoxify ROS has an impact on their radiosensitivity (22, 33). Research has shown that increased expression or activity of antioxidant enzymes was correlated with poor radioresponse (34–36). Upregulation of the ROS gene set (genes that are upregulated by ROS, meaning genes activated by oxidative stress or responsible for antioxidant activity) was associated with better OS in CESC patients (**Figure 2E**, p = 0.0056), while upregulation had a negative association with OS for BRCA patients (**Figure 2F**, p = 0.037).

The three last gene sets were related to DNA damage and in what way cancer cells can respond. DNA is the primary target of radiation and its damages are the prime source of the biological effects of radiation (24, 37, 38). UV response (down and up) involved the downregulated or upregulated genes after cells were radiated with UV radiation. The DNA repair gene set incorporates the genes involved in DNA repair. The upregulation of the UV Response Down gene set was associated with worse survival in CESC (**Figure 2G**, p= 0.001). For HNSC patients, the upregulation of the UV Response Up gene set was associated with worse OS (**Figure 2H**, p =0.0039) Neither upregulation nor downregulation of the DNA Repair gene set was associated with OS in any of the three cancer types.

To summarize the generated data per gene set, we calculated the cox proportional hazard ratios for every gene set (**Figure 2I**). The hazard ratio describes the probability of death of a patient, while the KM curves estimate the survival function. For the KM, a log rank test is used to test the hypothesis that different populations’ survival curves do not differ. The upregulation of the hypoxia gene set in HNSC was associated with an increased risk of death (**Figure 2I**, HR: 1.77). Both upregulation and downregulation of the G2M gene set were associated with a reduced risk of death in HNSC patients (**Figure 2I**, HR-up: 0.58; HR-down: 0.24). The upregulation of the ROS gene set was associated with a higher risk of death in BRCA (HR: 1.87) but displayed a reduced risk of death in CESC patients (HR: 0.41) (**Figure 2I**). CESC patients with an upregulation of the UV Response Down gene set were associated with three times higher risk of death (**Figure 2I**, HR: 3.06) and HNSC patients with an upregulation of the UV Response Up gene set were associated with a higher risk of death (HR: 1.80) (**Figure 2I**). In line with the KM curves, neither upregulation nor downregulation of the DNA repair gene set were associated with any hazard ratio.

### Metabolism Category

The metabolism category exist of seven gene sets: Glycolysis, Oxidative Phosphorylation (OXPHOS), Fatty Acid Metabolism, Cholesterol Homeostasis, Heme Metabolism, Xenobiotic Metabolism and Bile Acid Metabolism. Metabolic reprogramming of cancer cells is considered one of the hallmarks of cancer (39, 40). Increasing evidence suggests that metabolic reprogramming in cancer is one of the major factors contributing to radioresistance (41–43).

**Figure 3:**
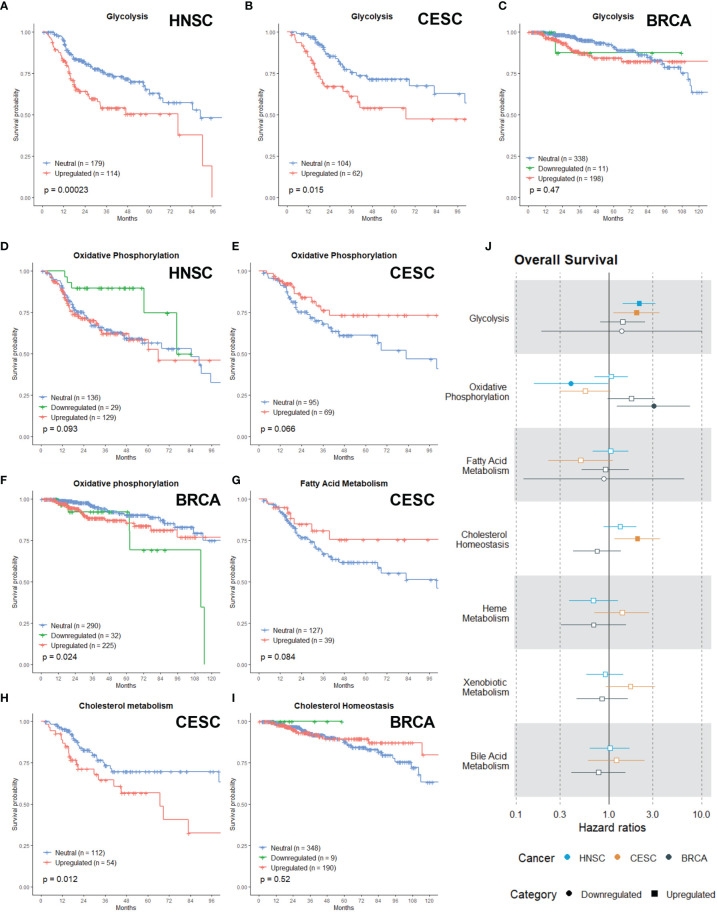
Associations of gene sets of the Metabolism category with patient overall survival times. Kaplan-Meier plots for the Glycolysis gene set of **(A)** HNSC, **(B)** CESC and **(C)** BRCA patients associated with OS. Kaplan-Meier plots for the Oxidative Phosphorylation gene set of **(D)** HNSC patients, **(E)** CESC patients and **(F)** BRCA patients associated with OS. **(G)** Kaplan-Meier plot for the Fatty Acid gene set for CESC associated with OS. Kaplan-Meier plots for the Cholesterol gene set for **(H)** CESC patients and **(I)** BRCA patients associated with OS. **(J)** Forest plot showing the results from CoxPH model fits for OS of all the Metabolism category gene sets. Results for the HNSC patients are in blue, brown for the CSC patients and black for the BRCA patients. Upregulated gene sets are depicted with a □ symbol [closed ■ means these results are significant (p>0.05)]. Downregulated gene sets are depicted with a ○ symbol [closed ● means these results are significant (p>0.05)].

Alterations in the glycolytic metabolism in cancer influences radioresponses and extensive research has been performed to develop molecules or inhibitors of several glycolytic targets (44, 45). In our analysis, the upregulation of the Glycolysis gene set was associated with a worse OS in HNSC (**Figure 3A**; p =0.00023) and CESC (**Figure 3B**; p=0.015), while no association was found for BRCA patients (**Figure 3C**; p = 0.47).

Although rewiring energy metabolism in cancer is mostly associated with a switch from OXPHOS to glycolysis (46), research demonstrates that cancer cells can use a wide range of energetic profiles and OXPHOS represents a major source of energy production (47). Recent evidence links the upregulation of OXPHOS or metabolic plasticity to increased radioresistance in oesophageal adenocarcinoma, breast, pancreatic and head and neck cancer (48–51). There appeared to be non-significant trend (in the first months) of better OS in HNSC when OXPHOS was downregulated (**Figure 3D**; p = 0.093), while in CESC there appeared to be a non-significant trend of better OS when OXPHOS was upregulated (**Figure 3E**; p =0.066). Downregulation of OXPHOS in BRCA patients was associated with worse OS (**Figure 3F**; p =0.024). Many cancers show an upregulated lipogenesis (52) and recent research linked this with enhanced radioresistance in prostate cancer and breast cancer cells (53–56). Nonetheless, no significant association was found for the Fatty Acid Metabolism gene set in any of the cancer types, however upregulation appeared to be associated with better OS in CESC patients (**Figure 3G**).

Cholesterol is vital for the survival and growth of mammalian cells. It is a membrane constituent and a precursor to bile acids and steroid hormones, which can initiate and promote colon, breast and prostate cancers (57). Targeting cholesterol metabolism with statins in clinical studies for cancer patients suggested an added benefit for patient survival across various cancer types (58–63). In combination with radiation, statins can improve clinical outcomes *via* their radiosensitizing properties. However, the available clinical data is conflicting (64, 65). Here, upregulation of the Cholesterol Homeostasis gene set was associated with worse OS for CESC patients (**Figure 3H**; p= 0.012). For BRCA patients, no association was found (**Figure 3H**; p = 0.52)

The critical role of heme in mitochondrial respiration and ADP/ATP exchange presumably explains how heme plays a pivotal role in fueling tumor cells’ proliferation. However, only a small amount of research has been performed regarding the role of heme metabolism and radioresistance. It has been proven that expression of heme oygenase is linked with response to radiotherapy in nasopharyngeal carcinomas (66). In solid tumors, the extracellular and intracellular distribution of drugs exhibits a high degree of variability, is largely controlled by Drug and xenobiotic metabolizing enzymes (DXME) and influx and efflux systems that transport drugs into and out from cells. Expression of DXME within tumor cells is known to play a role in tumor cell survival and in tumor-specific absorption, distribution, metabolism, and excretion (ADME) of drugs (67). Cancer cell drug resistance or sensitivity is critically impacted by expression of DMXE within tumors. Understanding which specific DXME contributes to response to particular drugs will lead to better precision medicine (68, 69). However, no research has been done in combination with radiotherapy. Bile acids are physiological detergent molecules synthesized from cholesterol exclusively in the liver (70–72). Bile acids itself have been implicated in the development of hepatocellular, bile duct and colon cancer (73–75). Research has mainly been performed to develop novel therapeutic approaches to treat cholestasis and inflammation-related liver diseases (72, 76). Upregulation or downregulation of the Heme Metabolism, Xenobiotic and Bile Acid Metabolism gene sets had no significant difference in survival (Data not shown).

Hazard ratio’s are again in line with the Kaplan-Meier curves. The risk of death for HNSC (HR: 2.13) and CESC (HR: 2.0) patients with an upregulated Glycolysis gene set was enhanced (**Figure 3J**). Downregulation of the OXPHOS gene set was associated with a lower risk of death in HNSC patients (**Figure 3J**; HR: 0.39). In contrast, this was associated with a higher risk of death in BRCA patients (**Figure 3J**; HR: 3.06). For the Fatty acid gene set, no associations were found (**Figure 3J**). In CESC patients, the upregulation of the Cholesterol Homeostassis gene was associated with a higher risk of death (**Figure 3J**, HR: 2.04). Lastly, upregulation or downregulation of the Heme Metabolism, Xenobiotic and Bile Acid Metabolism gene sets had no significant difference in survival or risk (**Figure 3J**).

### Proliferation Category

The proliferation category incorporates gene sets that are involved in the cell cycle progression, nutrient signals necessary for proliferation and proper function of cells. The proliferation category contains six gene sets: E2F Targets, Mitotic Spindle, MTORC1 Signaling, Myc Targets V1, Myc Targets V2 and P53. We expected that tumors with an upregulated gene set from the proliferation category would exhibit a more aggressive phenotype with a worse prognosis.

**Figure 4:**
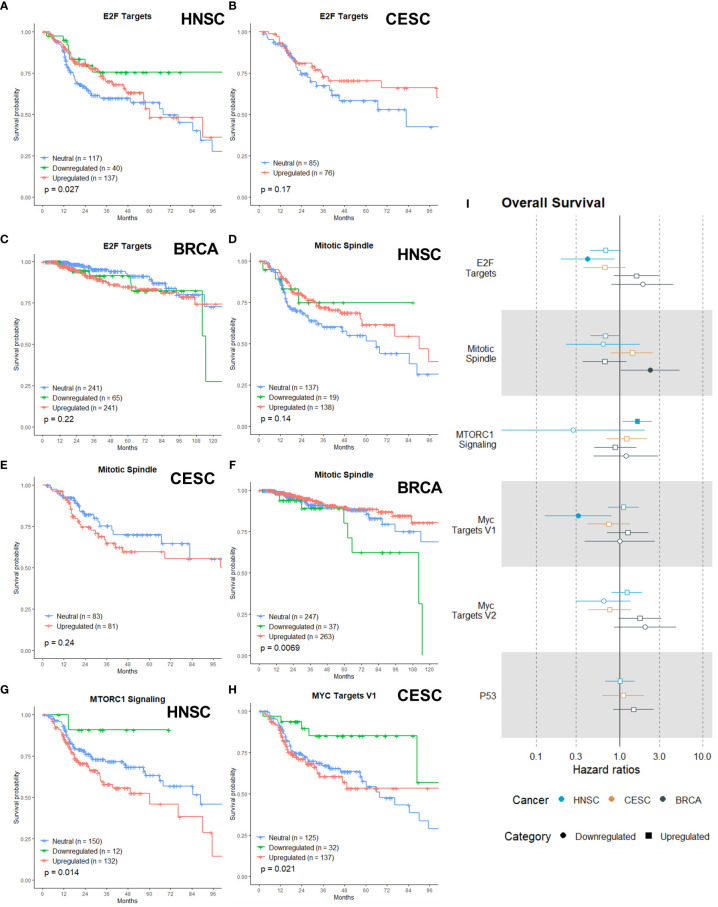
Associations of gene sets of the Proliferation category with patient overall survival times. Kaplan-Meier plots for the E2F Targets gene set of **(A)** HNSC, **(B)** CESC and **(C)** BRCA patients associated with OS. Kaplan-Meier plots for the Mitotic Spindle gene set of **(D)** HNSC patients, **(E)** CESC patients and **(F)** BRCA patients associated with OS. **(G)** Kaplan-Meier plot for the MTORC1 gene set for HNSC associated with OS. **(H)** Kaplan-Meier plot for the MYC Targets V1 gene set for CESC patients associated with OS. **(I)** Forest plot showing the results from CoxPH model fits for OS of all the Proliferation category gene sets. Results for the HNSC patients are in blue, brown for the CSC patients and black for the BRCA patients. Upregulated gene sets are depicted with a □ symbol [closed ■ means these results are significant (p>0.05)]. Downregulated gene sets are depicted with a ○ symbol [closed ● means these results are significant (p>0.05)].

E2Fs have emerged as major transcriptional regulators of cell cycle-dependent gene expression. E2F activity, as defined by expression of E2F target genes, is high in virtually all cancers, often owing to inactivation of its main binding partner and key regulator, RB (encoded by RB1), overexpression of cyclin-dependent kinases (CDKs) or inactivation of CDK inhibitors (77–79). The E2F family controls the transcription of cellular genes that are responsible for cell division (80). The expression pattern of E2F activators is abnormal in multiple human malignancies, such as ovarian cancer (81), breast cancer (82), bladder cancer (83), prostate cancer (84), lung adenocarcinoma (85) and colon cancer (86, 87). In conclusion, the E2Fs are a complex family of transcriptional regulators whose precise expression and activity are critical to protect cells from abnormal proliferation and cell cycle-generated genomic errors. So far, the E2Fs family has not yet been investigated as potential target for radiosensitization. Our analysis establish that downregulation of the E2F Targets gene set was associated with better OS in HNSC (**Figure 4A**; p =0.027). Although not significantly, we visually saw a trend of better OS with the upregulation of the E2F Targets gene of CESC patients (**Figure 4B**; p = 0.17) and no association was found in BRCA patients (**Figure 4C**; p=0.22).

The mitotic spindle is essential for cell division by mitosis, so inhibition is an effective way to delay or stop exit from mitosis. Anti-mitotic drugs like taxanes and vinca alkaloids were clinically effective as anti-tumor compounds (88, 89) and a lot more are under development (90). Some clinical trials have combined these newer drugs with radiotherapy, but the focus is on combination with chemotherapy (91). For both upregulation and downregulation of the Mitotic Spindle gene set visually there appeared to be a non-significant trend with better OS in HNSC patients (**Figure 4D**, p = 0.14), while for CESC patients visually there appeared a non-significant trend (in the first 60 months) with worse OS for the upregulation(**Figure 4E**, p =0.24). Downregulation of the Mitotic Spindle in BRCA was significantly associated with worse OS (**Figure 4F**, p=0.0069).

MTORC1 is key driver of cancer drug resistance, since it integrates a diverse set of environmental cues, from growth factor signals and nutritional status to direct metabolism and cell growth (92–95). Upregulation of MTROC1 signaling is also linked to enhanced radiotherapy resistance (96). Multiple groups have also demonstrated that the PI3K/AKT/mTOR pathway activation in response to radiotherapy is a principal mechanism of radioresistance (97, 98). Our data showed that upregulation of MTORC1 Signaling gene set was associated with worse OS in HNSC patients (**Figure 4G**, p = 0.014)

MYC is a master transcriptional regulator that controls almost all cellular processes. There is a wealth of data indicating that the deregulation of MYC activity occurs in many cancers and contributes to disease progression, metastatic potential, and therapeutic resistance (99, 100). Overexpressed c-Myc has also been found to promote radioresistance (101, 102). Only downregulation of the MYC Targets V1, and not MYC Targets V2, was associated with better OS in CESC patients (**Figure 4H**, p = 0.021) MYC Targets V2 was not associated with OS in any of the three cancers (data not shown).

The transcription factor P53 is known as a key molecule for determining cellular responses to ionizing radiation by initiating a spectrum of cell-type specific responses, including cell cycle arrest, senescence, apoptosis and DNA damage repair (103, 104). It has been shown that p53 determines tissue-specific radiosensitivity and mutations of p53 can influence this radiosensitivity (105). Surprisingly, neither upregulation nor downregulation was associated with OS in any of the three cancers (data not shown).

**Figure 4I** shows the calculated HRs. In HNSC patients, the downregulation of the E2F Targets gene set was associated with a lowered risk of death by more than two-fold (**Figure 4I**, HR: 0.41). No association was found for CESC or BRCA patients. Downregulation of the Mitotic Spindle in BRCA patients showed a two-fold higher risk of death (**Figure 4I**, HR: 2.3). The upregulation of the MTORC1 Signaling gene set was only associated with a higher risk of death in HNSC patients (**Figure 4I**, HR: 1.63). MYC Targets V1 was associated with a lower risk of death in CESC patients (**Figure 4I**, HR: 0.32), while MYC Targets V2 was not associated with risk of death in any of the three cancers (**Figure 4I**). Upregulation or downregulation of the P53 gene set was not associated with death risk in any of the three cancers (**Figure 4I**).

### Other Expression Sets With Clinical Relevance

The remaining categories of gene sets are the Development, Signaling, Cellular component and Pathway. Since only a small amount gene sets from the remaining categories have an association with OS in one of the three cancer types, we will not discuss all of them in detail but will only highlight the gene sets displaying an association with OS.

**Figure 5:**
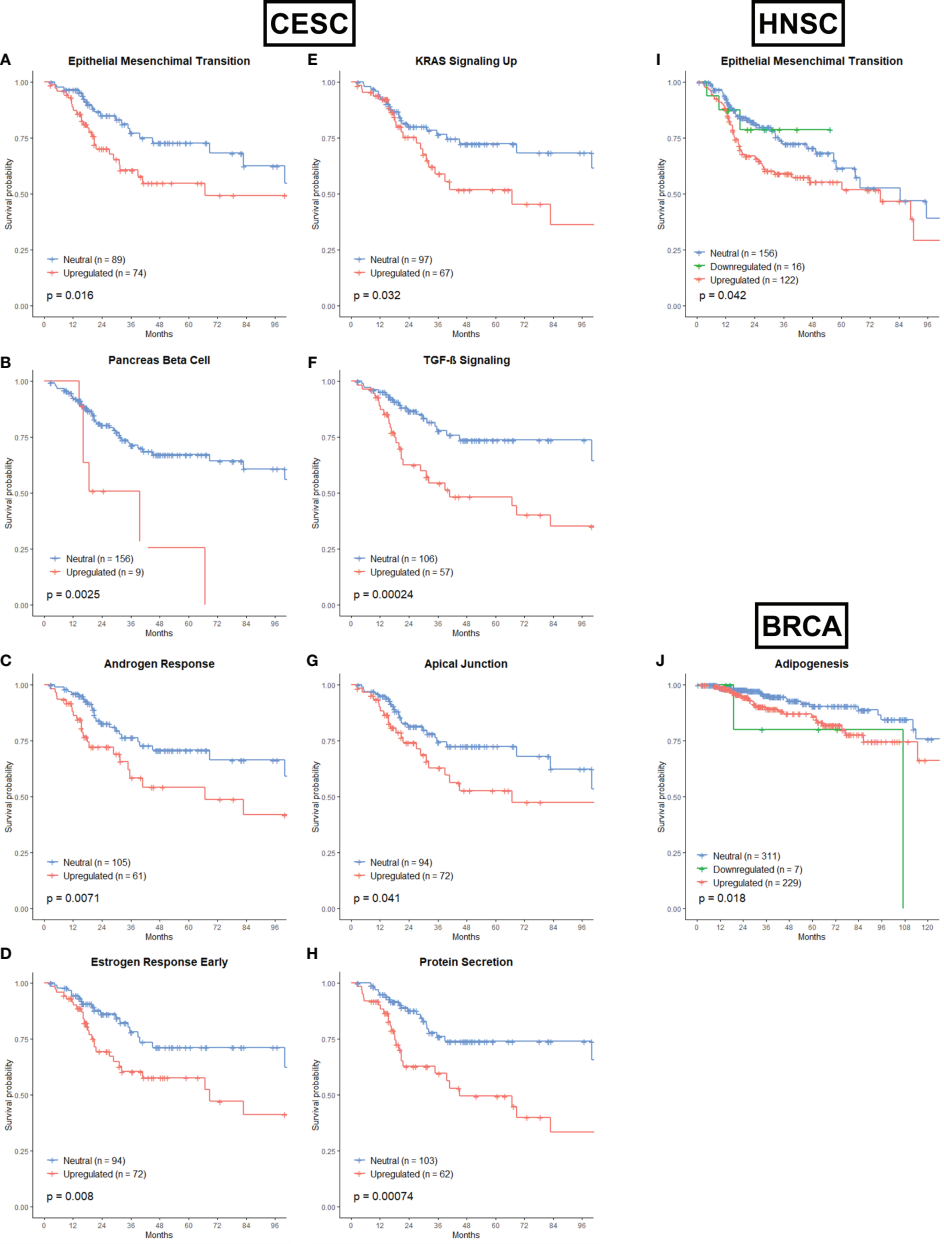
Associations of gene sets of the Development, Signaling, Cellular Component and Pathway categories with patient overall survival times. Kaplan-Meier plots of CESC patients associated with OS for **(A)** the Epithelial Mesenchymal Transition gene set, **(B)** the Pancreas Beta Cell gene set, **(C)** the Androgen Response gene set, **(D)** the Estrogen Response Early gene set, **(E)** KRAS Signaling Up gene set, **(F)** TGF-β Signaling gene set, **(G)** Apical Junction gene set and **(H)** Protein Secretion gene set. **(I)** Kaplan-Meier plot for the Epithelial Mensenchymal Transition gene set of HNSC patients associated with OS. **(J)** Kaplan-Meier plot for the Adipogenesis gene set of BRCA patients associated with OS.

Most of the associations between the remaining gene sets and OS were found in CESC patients (**Figures 5A**–**H**). From the Development category we showed that upregulation of the Epithelial Mesenchymal Transition gene set (**Figure 5A**, p = 0.016) and upregulation of the Pancreas Beta Cell gene set (**Figure 5B**, p=0.0025) were associated with worse OS. The epithelial-mesenchymal transition (EMT) is an important step leading to invasion and migration of various cancer cells (106). Recently, more and more evidence has shown that EMT functions as an essential process involved in radioresistance (107–109). The Pancreas Beta Cell gene set incorporates genes that were specifically upregulated in pancreatic beta cells. In the signaling category, the upregulation of the Androgen Response (**Figure 5C**, p = 0.0071), Estrogen Response Early (**Figure 5D**, p = 0.008), KRAS Signaling Up (**Figure 5E**, p = 0.032) and TGF-β Signaling (**Figure 5F**, p = 0.00024) were associated with worse OS. Androgens are expressed at different levels in men and women, and while they are important for proper development, they can also drive tumor growth. The role of the androgen receptor in prostate cancer has been extensively studied, but recent data suggest that androgen receptor signaling may also be important in breast cancer, glioblastoma, and additional tumor types (110, 111). One study found that significant subsets of gynecologic cancers express androgen receptors, which may have clinical relevance. Radiotherapy has been shown to induce Androgen receptor expression in prostate cancer cells, and androgen deprivation therapy sensitizes cancer cells to radiotherapy (112). Numerous studies have established a proof of concept that abnormal expression and function of estrogen receptors (ER) are crucial processes in initiation and development of hormone-related cancers and affect the efficacy of anti-cancer therapy (113). Research is ongoing to resolve the complex situation that impedes the therapeutic efficiency of endocrine therapy and radiotherapy. The KRAS Signaling Up gene set incorporated the genes that were upregulated by KRAS activation. KRAS mutations have been linked to cellular and clinical radioresistance (114–119). However, KRAS mutant tumors comprise a heterogeneous group of cancers and reported mechanisms of cellular radioresistance appear highly variable, consistent with the notion of intertumoral heterogeneity (116, 120). In cervical cancer, the presence of KRAS mutations was an independent predictor of disease recurrence (121). Members of the TGF-β family are key regulators of embryonic development, tissue homeostasis, and regeneration, and their malfunction has been implicated in cancer, fibrosis, immune diseases and many other pathologies (122). TGF-β has been reported to be an endogenous, radiation-inducible radioresistance factor in some cancer cells while not affecting the radio-sensitivity in others. In addition, TGF-β also regulates the transcription of various target genes responsible for the pathological changes of late radiation damage in the non-tumour-bearing tissues of previously irradiated patients (123, 124). Only one gene set from the cellular component category is associated with OS. Upregulation of the Apical Junction gene set is associated with worse OS in CESC patients (**Figure 5G**, p = 0.041). The apical junction complex is a cell-cell adhesion system present at the upper portion of the lateral membrane of epithelial cells integrated by the tight junction and the adherens junction. Research is just starting to understand the importance and therapeutic potential of apical junction complex proteins and their role in the early and late stages of cancer (125). Also in the pathway category is only one gene set associated with OS for CESC patients. Upregulation of the Protein secretion gene set is correlated with worse OS (**Figure 5H**, p = 0.00074). The dysfunction of the secretory pathway is the cause of a variety of systemic and developmental diseases, like cancer, diabetes, Parkinson’s disease, and congenital neurodegenerative disorders (126–129).

Only the upregulation of the EMT gene set was associated with worse OS in HNSC patients (**Figure 5I**, p=0.042). For BRCA cancer, the upregulation and downregulation of the Adipogenesis gene set (from the Development category) was associated with worse OS (**Figure 5J**, p = 0.018). It has already been proven that abnormal adipocyte metabolism is linked with radioresistance, mainly in BRCA (130, 131).

### Immune and Stromal Scores

**Figure 6:**
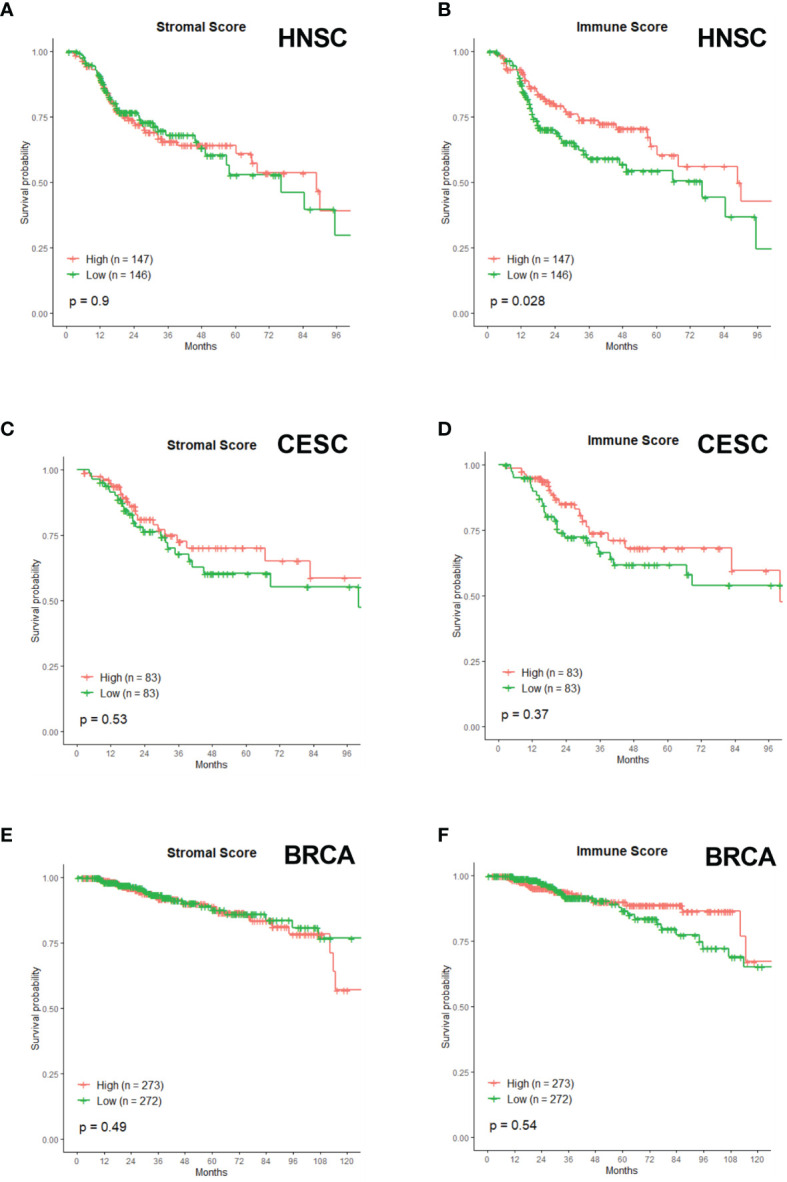
Associations of Stromal and Immune score with patient overall survival times. Kaplan-Meier plots of OS of HNSC patients with **(A)** Stromal Score and **(B)** Immune Score. Kaplan-Meier plots of OS of CESC patients with **(C)** Stromal Score and **(D)** Immune Score. Kaplan-Meier plots of OS of BRCA patients with **(E)** Stromal Score and **(F)** Immune Score.

In our analysis, none of the immune gene sets were associated with better prognosis or survival. This was very unexpected, especially since there is a known relationship between immune cells and RT outcome (132, 133). Therefore, we opted to investigate this further and correlated the infiltration of immune cells (or other cells) with survival after radiotherapy. Using the ESTIMATE algorithm (134), immune infiltration scores were calculated and patients were divided into high or low immune scores with the median as cut-off. Next, we compared the survival of the two groups (**Figures 6A, C, E**). A high immune infiltration was significantly associated with better OS in HNSC patients (**Figure 6A**), however this was not the case for CESC (**Figure 6C**) and BRCA (**Figure 6E**). Next to immune score, the stromal score was calculated using the ESTIMATE algorithm. This stromal score was developed to capture the presence of stromal cells in the tumor tissue. Again, no significant association of stromal score and OS was found for any of the three cancer types (**Figures 6B, D, F**).

## Discussion

More than 50% of all cancer patients are treated with radiotherapy at some point during their treatment. However, there is a lot of heterogeneity in the clinical responses to radiotherapy between different cancer types and even within the same cancer type. Resistance to radiotherapy is polymodal and associated with several biological alterations both within the tumor and the surrounding microenvironment. Radiosensitizers are needed to improve treatment response to radiation. Although the research into radiosensitizers already started 60 years ago, only a few radiosensitizers were implemented in the clinic. A wide range of obstacles, such as cancer stem cells, tumor heterogeneity, angiogenesis and vasculogenesis, metabolic alterations, drug-related adverse events, … poses a significant challenge in increasing the efficacy of RT by radiosensitizers (7, 135, 136). In this study, we attempted to unravel potential radiosensitization targets in a more systematic and clinically relevant way. Based on HNSC, CESC and BRCA patient cohorts that underwent radiotherapy and their parallel mRNA data, we investigated 50 hallmark gene sets that describe essential processes and pathways and their impact on patient survival.

We anticipated four of the eight gene set categories to be highly predictive with regard to OS in patients who underwent RT, namely the Radiobiological, Metabolism, Proliferation and Immune category. The six gene sets from the Radiobiological category were chosen to represent known biological processes that influence cancer cells’ radiosensitivity. Indeed, we observed that Hypoxia, G2M checkpoint and ROS gene sets were associated with OS in at least one of the three cancer types. Surprisingly, the other three gene sets related to cancer cells response to RT were overall not associated with OS. In recent years, researchers discovered that the metabolism of the cells also influences radiosensitivity and radioresistance. We observed that both Glycolysis and OXPHOS were associated with OS for one of the three cancer types. Extensive research has already been performed in developing molecules or inhibitors for several targets of glycolysis or OXPHOS and several clinical trials underway (40, 47). An interesting finding was the upregulation of the Cholesterol gene set associated with OS in CESC patients. The Cholesterol pathway has been successfully targeted with statins, however combinations with radiotherapy resulted in conflicting results (64), which should be further unravelled.

We expected that the gene sets from the Proliferation category would also be associated with OS in RT patients. These gene sets are involved in the cell cycle progression, nutrient signals necessary for proliferation, and cells’ proper function. We observed that downregulation of the E2F Targets was associated with better OS in HNSC. E2Fs are a complex family of transcriptional regulators and have not been investigated as a potential target for radiosensitization. Downregulation of the Mitotic Spindle gene set was associated with worse OS in BRCA. Several drugs are under development that target the mitotic spindle pathway. However, few have been tested in combination with radiotherapy (91). MTORC1 is a master regulator of cell growth and proliferation. Upregulation of the MTORC1 Signaling gene set was associated with worse OS in HNSC patients. Upregulation of the MTORC1 pathway has already been linked to enhanced RT resistance (96). MYC is a known oncogene and overexpressed c-MYC has been shown to promote radioresistance (101, 102). We observed that downregulation of the MYC Targets V1 was associated with better OS in CESC patients. Surprisingly, the P53 pathway was not correlated with OS in any of the three cancer types. It would be interesting to correlate outcomes of different gene sets with the expression of ki67 on the biopsies of the different tumors; unfortunately, this data is not available in the used databases. Ki67 is mainly expressed in actively proliferating cells and is a proliferation-related nuclear antigen (137). Ki67 has been widely investigated as a potential prognostic maker of proliferation in retrospective studies of malignant diseases (138–141).

Several other gene sets from the remaining categories (Development, Signaling, Cellular Component and Pathway) were also associated with OS. In CESC patients, the EMT, the Pancreas Beta cell, Androgen Response, Estrogen Response Early, KRAS Signaling UP, TGF-β Signaling, Apical Junction and the Protein secretion gene set was associated with OS. It is worth noting that the association of Androgen Response and Estrogen Response Early with OS in CESC patients was to be expected. Already in the 1970s, researchers linked sex hormones to genital cancer (142, 143). Several epidemiological studies have shown that multiple pregnancies and recent oral contraceptive use are potential cofactors of CESC (144). In addition, it was demonstrated that circulating levels of sex steroid hormone testosterone and possibly estradiol were positively involved in CESC (143, 145–147). Since androgens and estrogens are biochemically closely related, it is difficult to obtain solid evidence on whether they are separately or cooperatively involved in cervical carcinogenesis (145). For HNSC patients, only the upregulation of EMT gene set was associated with worse OS. For BRCA the upregulation and downregulation of the Adipogenesis gene set were associated with worse OS.

The most surprising results from this study occurred within the Immune category, where none of the examined gene sets were associated with better survival after RT. Merely a high immune score, a score for infiltrating immune cells, within HNSC correlated with better survival than a low immune score. The success of cancer immunotherapy has demonstrated that immune cells can be harnessed to eliminate tumor cells (148). It is becoming critical to understand the immune infiltration in the TME in the immunotherapy era to boost anti-tumor immunity further (131, 135). Furthermore, it has been shown that immune contexture (the density, functional orientation and location of immune cells) of the tumors is import for prediction of clinical outcome (149–152). Studies reported that HPV-related HNSC exhibited an increased immune infiltration in general compared with HPV-unrelated tumors (153). In CESC patients, tumor infiltration proved to be a superior prognostic factor compared to stromal lesions (154–156). Historically, BRCA has been regarded as an immunologically “cold” phenotype, however recent studies suggest potential in immunotherapeutic approaches to improve outcomes of specific subsets of BRCA patients (148, 157). RT has long been hypothesized to have actions complementary to those of immune checkpoint blockade, and a growing body of evidence indicates that cancer immunotherapy may also have radiosensitizing effects, which would provide unique benefits for loco-regional treatments (132). As a side note, we have to mention that no patient within our data set received CLTA4 or PD-1/PD-L1 inhibitors. The recent clinical success of these immunotherapies in different cancers suggest that these results should be interpreted with caution (158). It is possible that treating patients with immunotherapeutics could change the immune TME and lead to a better correlation with outcome. Nonetheless, in our study, we failed to show a survival benefit for any immune-related pathways but did demonstrate a better survival with a high immune score in HNSC.

Numerous research groups have used genomic-based approaches for the prediction of tumor response after RT. Most of these groups utilized high-throughput RNA expression technologies to develop gene expression signatures prognostic of low local recurrence risk or predictive of response to radiation treatment in the adjuvant setting for patients with early-stage disease. The initial studies where a correlation was described between gene expression and radiosensitivity were conducted *in vitro* on the NCI-60 panel of cancer cell lines. The best known are the studies where signatures of radiation sensitivity were identified using survival after 2Gy (SF2) or 8Gy (SF8) as a metric (159, 160). The group using the SF2 later developed the radiosensitivity index (RSI), which has been assessed in the clinic in various disease types, with varying levels of utility identified (161–165). Similar approaches were applied to identify radiosensitivity signatures and test them in Omnibus datasets or TCGA to create signatures for breast, head and neck, prostate, lung and glioma (166, 167).

In the latter cancer type, all patients receive radiotherapy as part of their treatment plan. As a consequence, the impact of radiotherapy on gene signatures has been extensively studied. Transcriptomic, methylation, mutational and mesenchymal signatures following radiotherapy all have been reported to correlate with patient’s prognosis in glioblastoma (GBM) (168–172). For example, gene signatures focusing on mesenchymal traits, which can be induced by radiation, distinctively correlate with worse prognosis (173). Within this present study, upregulation of the Epithelia Mesenchymal Transition gene set was only correlated with a worse prognosis in CESC patients, highlighting the importance of distinct approaches for each tumor type, and even tumor subtype. Additionally, GSEA was performed in an Omnibus GBM cohort and several cancer related pathways, such as p53 signaling pathway were enriched in the group with low OS (172).

The best-known genetic signature to predict radiation sensitivity across several different types of cancer is the panel of Torres-Roca et al. (174), which is based on the RSI. His team developed a calculation, called GARD (genomic-adjusted radiation dose), that uses the ten-gene panel to work out the biological dose based on an individual’s radiation sensitivity. Other groups have used genomic studies to compare patient-matched pre-RT and post-RT tumors to obtain insight into clonal evolution in response to treatment and understand radioresistance mechanisms (175). For example, whole-exome sequencing before and after chemoradiotherapy showed that co-occurring KRAS/TP53 mutations in rectal cancers conferred a poor response, confirming the radioresistance associated with this genotype (116, 176, 177). However, none of these signatures have been implemented into standard clinical settings and only give limited inside into the biological processes that underline radioresistance.

Our study contained several limitations. First, our research was based on gene expression of a single time-point. Gene expression analyses provide only a snapshot in time of the overall growth and treatment of the cancer. Keeping this in mind, the association between a particular gene set and survival can serve as a prognostic biomarker, even if this association neglects the influence of radiotherapy. A second limitation consists of a potential bias from variation in follow-up information in the TCGA database due to the retrospective nature of the TCGA cohort. Furthermore, in large public databases, such as TCGA, many sequenced disease states and settings do not include patients who received RT or offer adequate details concerning delivered RT schedules. Thirdly, is it possible that some gene expression profiles such as the metabolism or proliferation category not only correlate with radioresponse but also with the aggressiveness of the tumor. For example, it is well-established that metabolic reprogramming is linked with accelerated growth and proliferation of cancer cells (39, 178). Additionally, tumor aggressiveness between the three cancer types varies immensely. BRCA cancer patients, on average, display a more positive prognosis than HNSC or CESC patients. Better survival leads to fewer statistical events, which can influence the correlation with the investigated gene sets. Fourthly, all data is derived from patients receiving radiotherapy in an adjuvant setting post-surgery, potentially influencing the reported prognostic associations. Ideally, inclusion of data on neoadjuvant radiotherapy would be of great interest to complete this study. Sadly, many genomics studies lack radiation therapy treatment and outcome data, especially from neoadjuvant treatment, seriously limiting the data’s clinical utility (179). Lastly, we studied all genes separately. It can be expected that interactions between the different gene sets may play a role as well. We are currently developing a dedicated random subspace decision forest analyses. This supervised learning algorithm would be able to select individual genes and specific interactions and thereby improve the prediction accuracy (180). Our study found that none of the 50 hallmark gene sets were associated with OS for all three cancer types simultaneously. We believe that part of this heterogeneity can be explained by the above-described limitations.

However, our study’s heterogeneity across the three tumor types also highlights the necessity to invest in more personalized treatment. The old paradigm of one-size-fits-all cannot apply across and intra-cancer types in this era. The need for a diverse array of diagnostic and therapeutic options employed in oncology reflects cancer heterogeneity in general. To optimize personalized precision medicine, it is essential to understand the complexity of the underlying interactions between biological tissue and RT. On the one hand, clinical studies including RT that pro-actively investigate different omics (molecular, metabolic and imaging data) are needed to capture relevant data. On the other hand, more advanced modelling techniques become necessary to predict radiotherapy responses in order to understand the underlying trends across populations. Coupling the relevant data with novel modeling techniques such as machine learning will enhance our capabilities to establish a tailored precision treatment scheme per patient, where drug – radiotherapy combinations are put central.

To conclude, we have set up a clinically relevant approach using OS of HNSC, CESC and BRCA patients that underwent radiotherapy to associate with upregulation or downregulation of biological relevant hallmark gene sets. We established that 3 of the eight gene set categories, namely the Radiobiological, Metabolism and Proliferation, had predictive associations between several gene sets and OS. Surprisingly, we did not observe any associations between immune gene sets and OS in these patients cohorts. Interesting to note was the high heterogeneity across the three cancer types, which partly can be explained by the limitations of this study. However, this heterogeneity demonstrates that we need to opt for a tailored precision treatment scheme based on omics data. We believe that our study is the first step in this direction by using biologically relevant gene sets instead of single genes. However, there is a need for more databases or prospective studies that collect data from patients undergoing RT in a neo-adjuvant setting and capture RT outcome instead of survival. These databases or studies should go hand in hand with more complex modelling efforts to capture the complexity and interactions between the tumor and RT. Although we realise that a lot of work is still necessary, we believe this work can contribute to personalization of cancer treatment with regards to RT.

## Materials and Methods

### Gene Expression and Clinical Data

mRNA expression (RNA Seq V2 RSEM) and associated clinical data were obtained from cBioPortal (181, 182) of all the HNSC (523), CESC (297) and BRCA (1084) patients of the TCGA PanCancer Atlas. The mRNA expression data were downloaded in the form of z-score-transformed data. We extracted patients who underwent radiotherapy using the R package “dplyr” and “stringr” from the clinical data. In total 294 HNSC, 166 CESC and 549 BRCA patients underwent radiotherapy. All patients underwent surgery followed with adjuvant (chemo)radiotherapy. Data on radiation scheme and dose were downloaded from the PanCancer Atlas website (183).

### MsigDB Hallmark Collection

The molecular signature database (MsigDB) hallmark collection exists out of 50 hallmark gene sets. Hallmark gene sets summarize and represent specific, well-defined biological states or processes and display coherent expression. These gene sets were generated by a computational methodology based on identifying gene set overlaps and retaining genes that display coordinate expression. The hallmarks reduce noise and redundancy and provide a better delineated biological space for GSEA. Originally these 50 hallmark gene sets were divided into eight process categories: Cellular Component, Development, DNA damage, Immune, Metabolic, Pathway, Proliferation and Signaling. These were reordered into: Radiobiological, Metabolic, Proliferation, Development, Signaling, Cellular component, Pathway and Immune (**Supplementary Table 1**).

### Gene Set Expression Subtype Classification

We used the algorithm developed by Peng et al. (13) to classify individual tumors given the gene sets of the MsigDB Hallmark collection (9). For a specific patient, the classification was based on the deviation extent of the expression level of genes in a hallmark gene set from the cohort’s average values relative to other genes. GSE pre-ranked analysis was used to determine whether the genes from a hallmark gene set were enriched at the top or bottom of each sample’s z-ranked gene list. The GSEA algorithm (14, 15) was used from the publically available software (GSEA version 4.1.0). For a specific gene set, a tumor sample was classified into one of three distinct groups at FDR > 0.25: “upregulated”, “downregulated,” or “neutral”.

### Immune and Stromal Score

Stromal and immune scores were calculated with the ESTIMATE (estimation of stromal and immune cells in malignant tumor tissues using expression data) algorithm (134). ESTIMATE is an algorithm that uses gene expression signatures to infer the fraction of stromal and immune cells in the tumor samples. The stromal and immune scores predict the level of infiltrating stromal and immune cells in tumor tissue. We downloaded the Stromal and immune scores from the ESTIMATE website (184) of the HNSC, CESC and BRCA patients studied in our research. According to stromal and immune score, all the samples were divided into high and low groups separately.

### Survival Analysis

We evaluated whether the different gene set expression subtypes were associated with the patient’s overall survival time. Survival distributions were visualized using Kaplan-Meier curves and univariate Cox PH model plotted on a forest plot. The significance of the difference between the survival curves was assessed using a log-rank test. Significance for the Cox PH regression was determined using Wald’s test. Groups with five or fewer patients were excluded from the survival analysis. Survival analysis was performed using the computer program R (185) and the packages “survival”, “survminer”, “ggforestplot”, “tidyverse” and “ggplot2”.

## Data Availability Statement

Publicly available datasets were analyzed in this study. This data can be found here: https://www.cancer.gov/about-nci/organization/ccg/research/structural-genomics/tcga.

## Author Contributions

Conceptualization, data curation and investigation, SM. Supervision, ID and MR. Writing-original draft, SM. Writing – review and editing, ID and MR. All authors discussed the results and contributed to the final manuscript.

## Funding

This work was supported by the strategic research program “Societal Benefit of Markerless Stereotactic Body Radiotherapy: a Statistical Support based on Quantitative Imaging” (Zwaartepunt, SRP 53, 2019 - 2024) of the research council of the Vrije Universiteit Brussel.

## Conflict of Interest

The authors declare that the research was conducted in the absence of any commercial or financial relationships that could be construed as a potential conflict of interest.

## Publisher’s Note

All claims expressed in this article are solely those of the authors and do not necessarily represent those of their affiliated organizations, or those of the publisher, the editors and the reviewers. Any product that may be evaluated in this article, or claim that may be made by its manufacturer, is not guaranteed or endorsed by the publisher.
