# Supplementary material for: Radioresistance of Human Cancers: Clinical Implications of Genetic Expression Signatures
Source: Front Oncol. 2021 Oct 27;11:761901. doi: 10.3389/fonc.2021.761901 (PMC8579106; doi:10.3389/fonc.2021.761901)

**Supplementary Information**

**Radioresistance of human cancers: clinical implications of genetic expression signatures**

**de Mey et al.**

**Supplementary table 1: Gene set names, categories and descriptions.**


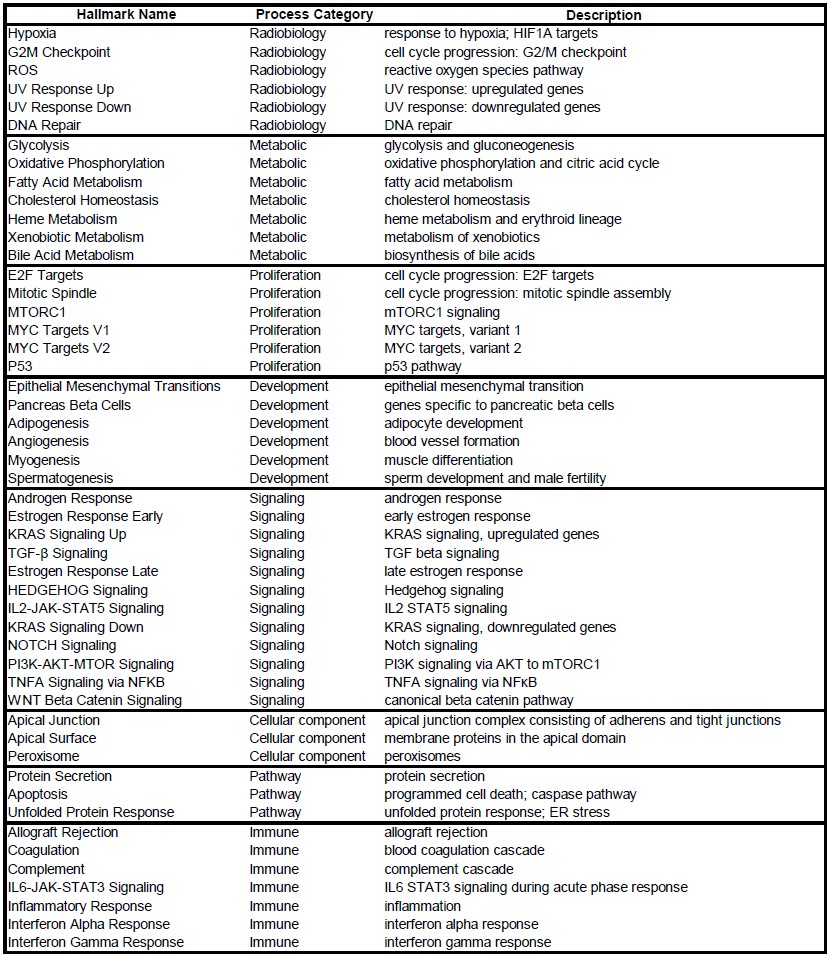

Supplement: Supplementary file 1 [file Table_1.docx]
